# The Social Attitudes Towards the Booster Dose of the COVID-19 Vaccine and the Associated Factors Among Residents of Riyadh, Saudi Arabia

**DOI:** 10.7759/cureus.46556

**Published:** 2023-10-05

**Authors:** Khaleel Alyahya, Wareef Y Almousa, Lama F Binsalamh, Ghadeer A Alturaifi, Lama H Alabdely, Norah F Aljulaihim, Layan M Aldosari

**Affiliations:** 1 Department of Anatomy, King Saud University College of Medicine, Riyadh, SAU; 2 College of Medicine, King Saud University Medical City, Riyadh, SAU

**Keywords:** vaccine literacy, associated factors, vaccination, covid-19, booster dose

## Abstract

Background

As a result of the Coronavirus (COVID-19) pandemic, global health was significantly affected. Therefore, the booster dose was approved to be administered to people who had completed a primary vaccination series in order to enhance their immunity. This study aims to identify the factors that lead to willingness or hesitancy toward the third/booster dose of the COVID-19 vaccine, to estimate the rate of acceptance and hesitancy toward the third/booster dose of the COVID-19 vaccine, and to measure third/booster dose COVID-19 vaccine literacy among residents of Riyadh.

Methods

This study is a quantitative analytical cross-sectional, questionnaire-based study from March 2022 to December 2022. The data were gathered using a convenience sampling technique from 435 participants in the Riyadh region 16 years old and above by using a validated questionnaire.

Results

Among the participants, 72.6% were females; 53.4% of young participants aged 16-25 years had a good knowledge of the booster dose versus 26.2% of those aged 45 years or above, with reported statistical significance (P=0.001). The functional literacy of the COVID-19 vaccine which is defined as the ability to read and write effectively was higher among the non-hesitant group compared to the hesitant group. The interactive/critical literacy of the COVID-19 vaccine, which is defined as the advanced abilities that enable people to make sense of information so they may take decisions that are relevant to their own lives, was higher among the non-hesitant group compared to the hesitant group. 72.2% of the study participants reported that if the booster dose of the COVID-19 vaccine was not mandatory by the government, they would not have taken it. Also, 19.1% thought that taking the booster dose of the COVID-19 vaccine would endanger their lives.

Conclusion

The findings of the current study revealed that the factors leading to the willingness or hesitancy toward the booster dose of the COVID-19 vaccine include age, gender, and side effects. Also, measuring the COVID-19 vaccine literacy revealed that it is higher among the non-hesitant group than the hesitant group although it was statistically insignificant. Meanwhile, further studies should be done to track and measure COVID-19 vaccine literacy over time, and examine the factors associated with the booster dose of COVID-19 vaccine hesitancy for more validation and application.

## Introduction

The coronavirus (COVID-19) pandemic had a significant impact on global health [1]. It spreads quickly, infecting thousands of people worldwide and has been associated with widespread morbidity and mortality [2,3]. As of April 14, 2022, a total of 500,186,525 cases and 6,190,349 deaths of COVID-19 had been confirmed [4]. The COVID-19 vaccination has been gaining interest globally since the pandemic began in 2020 as a means of prevention [5]. The effectiveness of the first vaccine against the COVID-19 disease was proven for the first time on the date of December 11, 2020, by the Food and Drug Authority [6]. Since then, vaccination programs were rolled out globally. Initially, the vaccine was available only to 16-year-olds and above. After less than a year on May 10, 2021, the vaccine became available and safe for the age group of 12-15 years old [6]. With time, the immunity and clinical protection of the vaccine has fallen below a rate deemed sufficient in the vaccinated population [5].

Therefore, the administration of booster dose to those who had completed a primary vaccination series was approved in an effort to enhance the immunity against the viral disease [5]. Since December 19, 2021, efforts started by the Saudi Ministry of Health (MOH) urging the population to take a booster dose of COVID-19 [7]. The objective of a booster dose is to restore vaccine effectiveness from initial doses as well as to help people maintain strong protection against the antigen from severe coronavirus disease [8]. As a result, the booster dose of the COVID-19 vaccine has been shown to reduce the transmission and spread of the coronavirus [9]. Vaccine hesitancy is defined by the World Health Organization (WHO) as “the delay in acceptance or refusal of vaccination despite availability”, and has resulted in a global health threat with negative socioeconomic and health effects on individuals and their communities [10]. Despite the evidence of the effectiveness of the booster dose in enhancing immunity against the two variants Delta and Omicron, the receiving of the booster dose is still controversial [10]. The acceptance rates of the booster dose of COVID-19 varied based on several factors including age, occupation, gender, vaccination history, educational level, vaccine literacy, monthly household income, health status and governmental laws [10-13]. Females are at higher risk of being infected with COVID-19 [14]. Meanwhile, a study found that the mortality rate is higher for males, but respondents are not considered to be a complete representation of the attitudes of residents in other regions of Italy, as the survey was conducted in a single center [14].

This study focused on estimating the acceptance and hesitancy in a different geographical place and population which is Riyadh, the capital of Saudi Arabia, as well as investigating whether the preference of people to take the dose stems from complete confidence and conviction, or because of the government's laws that require the booster dose to enjoy personal interests such as traveling, working, and shopping. Therefore, it intended to assess societal vaccine literacy, as well as the factors that lead to the willingness or hesitancy toward the booster dose of COVID-19, to increase society's awareness of the booster dose of COVID-19 for future booster dose acceptance. This study attempted to close these gaps in the literature. So, this study could be able to contribute to the understanding of acceptability and hesitancy about taking the COVID-19 booster dosage. Hence, it could help to design strategies to overcome hesitancy and improve COVID-19 vaccine takeup.

## Materials and methods

Study design, setting and participants

A quantitative, cross-sectional analytical, questionnaire-based study was conducted in the Riyadh region from March 2022 to December 2022. The data for this study were collected between 22 August and 25 August 2022 using an online questionnaire to explore the social behavior toward the booster dose of the COVID-19 vaccine and the associated factors among residents of Riyadh, Saudi Arabia. This study targets subjects aged 16 or above who live in the Riyadh region.

Data collection

Data was collected from Riyadh residents who were 16 years old and above using a developed self-reported online questionnaire through Google Forms and added 12 items for COVID-19 vaccine literacy which was developed by Biasio et al. [15]. The questionnaire comprises 40 questions overlying the following five sections. The first section for sociodemographic information (six items) included age, gender, occupation/employment status, educational level, income, and chronic diseases. The second section for COVID-19 vaccine history (four items) included the vaccine type, and the number of doses. The third section for booster dose of COVID-19 vaccine hesitancy (seven items) and the fourth section for booster dose of COVID-19 knowledge (11 items) were both assessed on a five-point Likert scale: “Strongly Disagree = 1”, “Disagree = 2”, “Neutral = 3”, “Agree = 4”, “Strongly Agree = 5”. A scale that devolved by Biasio et al. was used for the fifth section to measure COVID-19 vaccine literacy which has already been validated (12 items) four items for the functional literacy which is defined as the ability to read and write effectively, and eight for the interactive/critical literacy which is defined as the advanced abilities that enable people to make sense of information so they may take decisions that are relevant to their own lives, assessed on a four-point Likert scale: “Never = 1”, “Rarely = 2”, “Sometime = 3” to “Often = 4” [15,16]. A pilot study has been performed to pre-test the study.

Sampling and sample size

A minimum target sample size of 435 was calculated using the single proportion equation (n = z α ^ 2 p (1-p) / d ^ 2), where p=0.62 which is the proportion of prevalence of the willingness to take the booster dose in recent research [11], d=5%, and Z for 95%=1.96; the result was 362.03. To account for a potential non-response rate of 20%, 20% of the sample size was added, resulting in a total sample size of 435.

Ethical considerations

The Institutional Review Board (IRB) of King Saud University approved this study on 16 August 2022. Participants were not obliged to complete the questionnaires and could withdraw at any point and no incentives or rewards were given to participants. Participants were assured of the anonymity and confidentiality of their information. This research did not receive any specific grant from funding agencies in the public, commercial, or not-for-profit sectors.

Statistical analysis

Statistical analysis was conducted using IBM SPSS version 21 (IBM Corp., Armonk, NY, USA); 5% was set as the significance level. Overall knowledge level regarding COVID-19 booster dose was assessed by summing up discrete scores for different correct knowledge items. The overall knowledge score was categorized as poor level if the participant's score was less than 60% of the overall score and good level of knowledge was considered if the participant's score was 60% or more of the overall score. Descriptive analysis was carried out using frequency distribution and percentages for study variables such as respondents' personal information, income level, and vaccination data. COVID-19 vaccine literacy among COVID-19 vaccine-hesitant and COVID-19 vaccine non-hesitant groups was compared using an independent-sample t-test. Pearson chi-square test for significance was used to perform cross-tabulation to show the distribution of participants' overall knowledge level by their socio-demographic data. Also, participants' willingness or hesitancy toward the booster dose of the COVID-19 vaccine was tabulated.

## Results

The study questionnaire was completed by 435 participants who fulfilled the inclusion criteria. Participants’ ages ranged from 16 years and above, with a mean age of 38.1 ± 13.6 years old. Out of 435 participants, 316 (72.6%) were females. As for employment status, 237 (54.5%) were unemployed, 173 (39.8%) were employed in the non-healthcare sector, and 25 (5.7%) were employed in the healthcare sector. There were 254 (58.4%) with bachelor’s degree, 123 (28.3%) high school or equivalent, and 38 (8.7%) postgraduate degree holders. Monthly income was low among 142 (32.6%) participants, but average among 225 (51.7%). A total of 50 (11.5%) were asthmatic, 48 (11%) had hypertension, 34 (7.8%) were diabetic, and 241 (55.4%) had no chronic diseases (Table 1).

**Table 1:** Socio-demographic data of study participants (n = 435)

Socio-demographic data	N	(%)
Age in years		
16-25	103	23.7
26-35	44	10.1
36-45	80	18.4
46-55	145	33.3
56+	63	14.5
Gender		
Male	119	27.4
Female	316	72.6
Occupation/employment status		
Unemployed	237	54.5
Governmental employees other than the healthcare sector.	116	26.7
Private sector other than healthcare sector	57	13.1
Employed in healthcare sector	25	5.7
Education level		
Less than high school	20	4.6
High school or equivalent	123	28.3
Bachelor’s degree	254	58.4
Postgraduate	38	8.7
Monthly income level		
Low income (< 6000 SR)	142	32.6
Average income (6000-20000 SR)	225	51.7
High income (> 20000 SR)	68	15.6
Chronic diseases		
None	241	55.4
Hypertension	48	11.0
Asthma	50	11.5
Diabetes Mellitus	34	7.8
Other chronic diseases	62	14.3

The functional literacy of the COVID-19 vaccine was higher among the vaccine non-hesitant group (M = 1.67, SD = 0.75) compared to the hesitant group (M = 1.52, SD = 0.95). The interactive/critical literacy of the COVID-19 vaccine was higher among the vaccine non-hesitant group (M = 2.74, SD = 0.69) compared to the hesitant group (M = 2.57, SD = 0.98), however, these results were statistically insignificant (Table 2).

**Table 2:** COVID-19 vaccine literacy among COVID-19 vaccine hesitant and COVID-19 vaccine non-hesitant groups

Variable Name	Received COVID-19 vaccine		p-value
	Yes (n = 425)	No (n = 10)	
Functional literacy	1.67 ± 0.75	1.52 ± 0.95	0.544
Interactive/critical literacy	2.74 ± 0.69	2.57 ± 0.98	0.455

Table 3 shows that 53.4% of young participants (16-25) had a good knowledge of the booster dose versus 26.2% of those aged 45 years or above, with reported statistical significance (P=0.001). Also, 49.6% of male participants had a good knowledge compared to 35.8% of females (P=0.009). Those with a post-graduate degree were also noted to have a good knowledge of the booster dose (50%) versus 25% of those with less than a high school degree (P=0.122).

**Table 3:** Factors affecting participants' knowledge regarding third/booster dose of the COVID-19 vaccine

Factors		Knowledge level				p-value
		Poor		Good		
		N	(%)	N	(%)	
Age in years	16-25	48	46.6	55	53.4	<0.001
	26-35	27	61.4	17	38.6	
	36-45	50	62.5	30	37.5	
	46-55	107	73.8	38	26.2	
	56+	31	49.2	32	50.8	
Gender	Male	60	50.4	59	49.6	0.009
	Female	203	64.2	113	35.8	
Occupation/Employment status	Unemployed	146	61.6	91	38.4	0.371
	Governmental employees other than healthcare sector.	70	60.3	46	39.7	
	Private sector other than healthcare sector.	36	63.2	21	36.8	
	Employee in healthcare sector.	11	44	14	56	
Educational level	Less than high school	15	75	5	25	0.122
	High school or equivalent	68	55.3	55	44.7	
	Bachelor’s degree	161	63.4	93	36.6	
	Post-graduate	19	50	19	50	
Chronic diseases	None	147	58.6	104	41.4	0.122
	Hypertension	34	70.8	14	29.2	
	Asthma	32	64	18	36	
	Diabetes Mellitus	17	50	17	50	
	Other chronic diseases	40	64.5	22	35.5	

A total of 72.2% of the study participants reported that if the booster dose of the COVID-19 vaccine was not mandatory by the government, they would not have taken it. Also, 54.9% thought that the first two doses of the COVID-19 vaccine protected them and there is no need for a booster dose, 48.7% believed there is a high probability that more side effects of the booster dose of the COVID-19 vaccine would be discovered which made them hesitant about taking it, and 42.5% reported that they cannot tolerate another dose of the COVID-19 vaccine because the side effects of the previous (first and second) doses were severe but only 19.1% thought that taking the booster dose of the COVID-19 vaccine would endanger their lives. On the other hand, 56.3% reported that they took the booster dose of the COVID-19 vaccine to protect their family from getting the COVID-19 infection (Table 4).

**Table 4:** Willingness or hesitancy toward the third/booster dose of the COVID-19 vaccine among residents of Riyadh, Saudi Arabia

Hesitancy/willingness	Strongly disagree		Disagree		Neutral		Agree		Strongly agree	
	N	(%)	N	(%)	N	(%)	N	(%)	N	(%)
I think there is an ability to discover more side effects of the third/booster dose of the COVID-19 vaccine in the upcoming years which made me hesitant about taking it.	24	5.5	61	14.0	138	31.7	87	20.0	125	28.7
If the third/booster dose of the COVID-19 vaccine was not mandatory by the government, I would not have taken it.	24	5.5	36	8.3	61	14.0	119	27.4	195	44.8
If I take the third/booster dose of the COVID-19 vaccine, there will be a danger to my life.	67	15.4	120	27.6	165	37.9	43	9.9	40	9.2
I took the third/booster dose of the COVID-19 vaccine to protect my family from COVID-19 infection.	44	10.1	58	13.3	88	20.2	138	31.7	107	24.6
I think the first two doses of the COVID-19 vaccine provided me with protection and there is no need for a third/booster dose.	29	6.7	40	9.2	127	29.2	115	26.4	124	28.5
I had a previous infection which enhanced the importance of taking the third/booster dose of the COVID-19 vaccine for me	138	31.7	112	25.7	101	23.2	57	13.1	27	6.2
I cannot tolerate another dose of COVID-19 vaccine (the first and second) because the side effects of the previous ones were severe.	57	13.1	79	18.2	114	26.2	84	19.3	101	23.2

## Discussion

Based on our knowledge, this is the first cross-sectional study that used a convenience sample of the population in Riyadh, Saudi Arabia, to evaluate public approval of receiving a booster dosage of the COVID-19 vaccine and its contributing factors. Socio-demographic factors showed different sample characteristics. A previous study done in Jordan by Al-Qerem et al. has found that demographic differences influence beliefs and acceptance of COVID-19 booster vaccination [17]. In this study, the majority of the respondents were females which could be linked to psychological gender differences. Previous studies also have shown that women have better compliance with public health policies during the COVID-19 pandemic as they are more likely to define it as a serious health problem [18].

A validated instrument has been used to determine the COVID-19 vaccine functional and interactive/critical literacy of the study participants [15]. Functional literacy is the ability to read and write effectively, whereas interactive/critical literacy refers to the advanced abilities that enable people to make sense of information so they may take decisions that are relevant to their own lives [16]. In this study, participants who accepted the booster dose scored higher on functional, interactive/critical literacy than those who were hesitant to get a booster dose. The association between vaccine literacy and vaccine hesitancy was measured using similar tools in one study and it was statistically significant that the functional literacy among vaccine non-hesitant group was higher than among the vaccine-hesitant group with a mean difference of −0.49, however, it was statistically insignificant in the current study [11].

This study looked at the socio-demographic factors and their association with the knowledge about the booster dose of COVID-19. There was a dominance of females at 74.6% of participants. However, males were found to have higher knowledge about the booster dose for COVID-19 at 49.6%, p=0.009. This was not found in a study conducted in the UAE where age and gender variables were insignificant. However, it was found that post-graduate educational status has higher knowledge about the booster dose at 72.6%, p <0.001, and employees in the health sector also have a higher knowledge at 54.9% p = 0.03 [19].

This study found age differences in knowledge about the booster of the COVID-19 vaccine favoring younger ages (16-25) at 53.4%, p=0.001. This was not demonstrated in studies done in Jordan, the United States, and China [11,12,17]. A possible explanation of this result can be due to the difference in the level of familiarity with the new media compared to traditional media among different age groups. Social media outlets were more effective in delivering messages about COVID-19 and preventive measures that include vaccination. Chronic diseases were not significantly associated with increased knowledge about booster doses. Meanwhile, in a study conducted in Ajman University, it was a crucial factor that encouraged people to take the booster dose [19]. It was noticed only a UAE study discussed the association between socio-demographic data and the knowledge level of the booster dose of COVID-19, and the results showed an association between the socio-demographic data and the knowledge of the booster dose [19]. This study showed that participants with a higher education level, with a prior history of COVID-19 infection, and a previous experience of taking the vaccine all have demonstrated more knowledge of the booster dose. Result differences between the studies could be due to the geographical, sample size, and sample characteristics variability, which indicates the need for more studies to be done for more validity.

Participants in a study done in Jordan claimed that the severity of the side effects and a mild infection with COVID-19 were additional reasons that probably kept them from getting a booster dosage of the COVID-19 vaccine [17]. In this study, 48.7% of participants thought there is a potential for more side effects from the booster dose of the COVID-19 vaccine which has made them unwilling to take it. Because of this, strategies are needed to be focused and consider the reasons that make people hesitant to receive the booster dose. These findings suggest that the main reported reason for not receiving a booster vaccine dose was the lack of solid scientific evidence about the advantage of the booster dose, followed by the perceived lack of benefit from a booster dose in the short term and the fear of potential side effects.

Regarding the protective motivation that drives people to get the vaccine, there is a study conducted in Fukushima that reported 81% believe that the main reasons for accepting the booster vaccination is related to infection control necessity, while 47.3% believe that vaccines are highly effective [20]. Also, a study found that China had the highest acceptance rate at 88.6%, and that nations with acceptance rates over 80% often had high levels of trust in their governments [21]. Furthermore, additional recent research claimed that the majority of those who did not obtain a booster dose showed less satisfaction with their initial COVID-19 vaccinations and that they lost trust in the Saudi Ministry of Health, the WHO, medical professionals, and the efficacy of all vaccines [21]. On the other hand, this study discovered that taking the booster dose increased significantly with government laws. However, prior studies have established a lack of knowledge regarding the influence of government laws on social behavior, especially in Saudi Arabia.

Limitations

The study's methodology relied on convenience sampling and an online questionnaire that participants completed themselves. Meanwhile, online surveys can provide a private environment for the respondents to finish the questionnaires honestly and accurately, thereby reducing any possible social desirability or interviewer biases. However, the access to the online survey that was used in this study was limited by the people who had access to the internet, and by those who were interested in this study. Although a comprehensive survey has been conducted, there remains a possibility of non-response bias, which may impact the reliability and generalizability of our findings. It was noticed that females were more acceptable and responsive to answer the questionnaire; so this study was limited by unequal distribution where females were dominant in the sample. Also, a history of side effects from previous doses and media preferences were important confounding factors that could not be eliminated.

Research implication

This study contributes to the understanding of acceptability and hesitancy about COVID-19 booster dosage. Hence, it can assist the Saudi Arabia government and relevant agencies to design more scientific and targeted roll-out strategies to overcome hesitancy and improve the booster dose of the COVID-19 vaccine uptake.

## Conclusions

In conclusion, the findings of the current study revealed that the factors leading to the willingness or hesitancy toward the booster dose of the COVID-19 vaccine include age, gender, and side effects. Also, measuring the COVID-19 vaccine literacy revealed that it is higher among the non-hesitant group than the hesitant group although it was statistically insignificant. Meanwhile, further studies should be done to track and measure COVID-19 vaccine literacy over time, and examine the factors associated with the booster dose of COVID-19 vaccine hesitancy for more validation and application. To improve the acceptance rate of vaccination among hesitant groups, there is a need for new regulations to be issued and run by the government and the public health authorities through educational framework to provide significant scientific information about the booster dose of the COVID-19 vaccine in order to minimize the misleading information spread online by non-official organizations.
